# Genome Signature Difference between *Deinococcus radiodurans* and *Thermus thermophilus*

**DOI:** 10.1155/2012/205274

**Published:** 2012-03-04

**Authors:** Hiromi Nishida, Reina Abe, Taishi Nagayama, Kentaro Yano

**Affiliations:** ^1^Agricultural Bioinformatics Research Unit, Graduate School of Agricultural and Life Sciences, University of Tokyo, Bunkyo-ku, Tokyo 113-8657, Japan; ^2^Bioinformatics Laboratory, Department of Life Sciences, School of Agriculture, Meiji University, 1-1-1 Higashi-Mita, Tama-ku, Kawasaki, Kanagawa 214-8571, Japan

## Abstract

The extremely radioresistant bacteria of the genus *Deinococcus* and the extremely thermophilic bacteria of the genus *Thermus* belong to a common taxonomic group. Considering the distinct living environments of *Deinococcus* and *Thermus*, different genes would have been acquired through horizontal gene transfer after their divergence from a common ancestor. Their guanine-cytosine (GC) contents are similar; however, we hypothesized that their genomic signatures would be different. Our findings indicated that the genomes of *Deinococcus radiodurans* and *Thermus thermophilus* have different tetranucleotide frequencies. This analysis showed that the genome signature of *D. radiodurans* is most similar to that of *Pseudomonas aeruginosa*, whereas the genome signature of *T. thermophilus* is most similar to that of *Thermanaerovibrio acidaminovorans*. This difference in genome signatures may be related to the different evolutionary backgrounds of the 2 genera after their divergence from a common ancestor.

## 1. Introduction

In the present bacterial taxonomic system, the extremely radioresistant bacteria of the genus *Deinococcus* and the extremely thermophilic bacteria of the genus* Thermus* belong to a common lineage with remarkably different characteristics [1, 2]. Comparative genomic analyses have shown that after their divergence from a common ancestor, *Deinococcus* species seem to have acquired numerous genes from various other bacteria to survive different kinds of environmental stresses, whereas *Thermus* species have acquired genes from thermophilic archaea and bacteria to adapt to high-temperature environments [3]. For example, the aspartate kinase gene of *Deinococcus radiodurans* has a different evolutionary history from that of *Thermus thermophilus* [4]. In addition, *D. radiodurans* has several unique protein families [5] and genomic characters [6], and there is no genome-wide synteny between *D. radiodurans* and *T. thermophilus* [7]. However, phylogenetic analyses based on both orthologous protein sequence comparison and gene content comparison have shown that the genomes of *Deinococcus* and *Thermus* are most closely related with each other [3, 8]. The trinucleotide usage correlations have been used to predict the functional similarity between two RecA orthologs of bacteria including *D. radiodurans* and *T. thermophilus* [9].

 If the genes acquired through horizontal gene transfers are different between *Deinococcus* and *Thermus*, then the genomic base composition (GC content) and/or genome signature can be hypothesized to also be different between these 2 genera. However, the GC content of *D. radiodurans* (67%) is similar to that of *T. thermophilus* (69.4%). The genome signature, on the other hand, is a powerful basis for comparing different bacterial genomes [11–19].

 Phylogenetic analyses based on genome signature comparison have been developed, and these analyses are useful for metagenomics studies [20]. It was reported that comparative study using the frequency of tetranucleotides is a powerful tool for the bacterial genome comparison [21]. In this study, we compared the relative frequencies of tetranucleotides in 89 bacterial genome sequences and determined the phylogenetic positions of *D. radiodurans* and *T. thermophilus*. 

## 2. Methods

### 2.1. Construction of Phylogenetic Relationships Based on the Relative Frequencies of Tetranucleotides in 89 Genome Sequences

 We compared the relative frequencies of tetranucleotides in the genome sequences. The frequencies of the 89 bacteria were obtained from OligoWeb (oligonucleotide frequency search, http://insilico.ehu.es/oligoweb/). The 89 bacterial species are part of a list that which covers a wide range of bacterial species published in a previous report [8]. Each frequency vector consisted of 256 ( = 4^4^) elements. The Euclidean distance between 2 vectors was calculated using the software package R (language and environment for statistical computing, http://www.R-project.org). On the basis of the distance matrix, a neighbor-joining tree was constructed using the MEGA software [10].

### 2.2. Ranking Based on Similarities between the Relative Frequencies of Tetranucleotides according to Correspondence Analysis

Correspondence analysis [22], which is a multivariate analysis method for profile data, was performed against the relative frequencies of tetranucleotides in 89 genomes. Correspondence analysis summarizes an originally high-dimensional data matrix (rows (tetranucleotides) and columns (genomes)) into a low-dimensional projection (space) [23, 24]. Scores (coordinates) in the low-dimensional space are given to each genome. The distance between plots (genomes) in a low-dimensional space theoretically depends on the degree of similarity in the relative frequencies of tetranucleotides: a short distance means similar relative frequencies of tetranucleotides between genomes, whereas a long distance means different relative frequencies. Thus, distance can be used as an index for similarity among genomes in the relative frequencies of tetranucleotides. Distances between all genome pairs were calculated, and then a ranking for distances was obtained.

## 3. Results and Discussion

**Figure 1:**
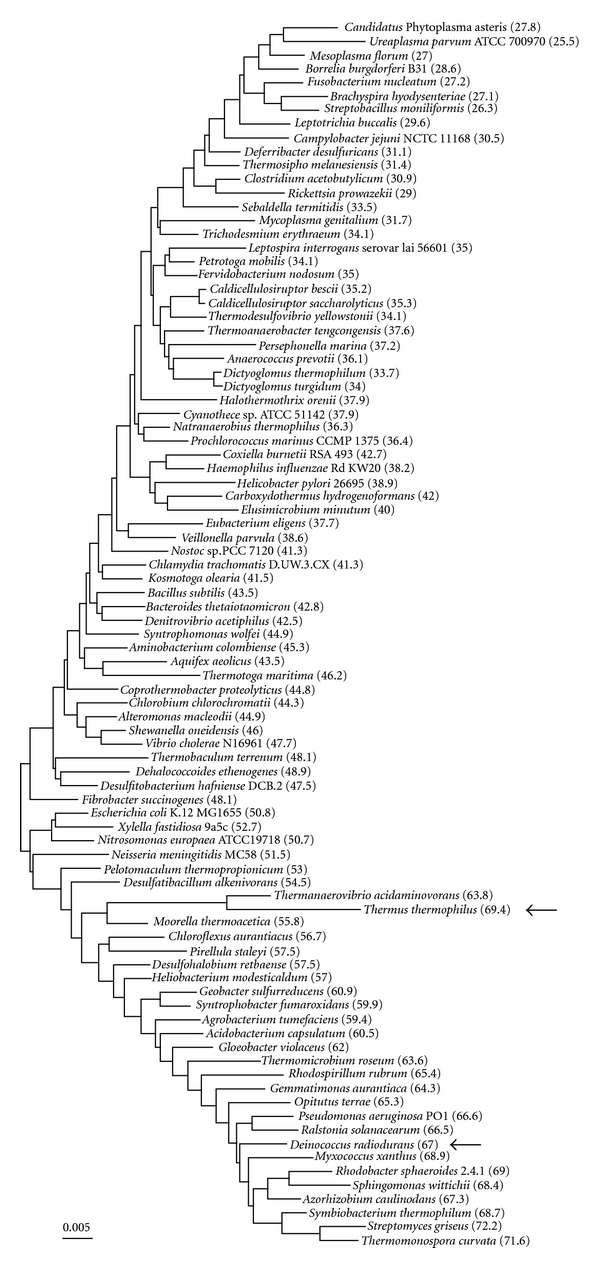
Neighbor-joining tree based on tetranucleotide sequence frequencies in 89 genomes. The frequencies for 89 bacteria were obtained from OligoWeb (oligonucleotide frequency search, http://insilico.ehu.es/oligoweb/). Each frequency vector consisted of 256 elements. The Euclidean distance between 2 vectors was calculated using the software package R (language and environment for statistical computing, http://www.R-project.org). On the basis of the distance matrix, a neighbor-joining tree was constructed using the MEGA software [10]. Numbers in parentheses indicate the GC content (percentage) of each genome sequence. Arrows indicate the positions of *Thermus thermophilus* and *Deinococcus radiodurans*.

In the neighbor-joining tree (Figure 1), *D. radiodurans* is located in the high-GC-content cluster, whereas *T. thermophilus* is grouped with *Thermanaerovibrio acidaminovorans *and their group is located away from the high-GC-content cluster. The neighbor-joining tree (Figure 1) was greatly influenced by the genomic GC content bias; most of the well-defined major taxonomic groups did not form a monophyletic lineage. This result indicates that each constituent of the well-defined major group has diversified by changing its genome signature during evolution. It is consistent with a previous paper indicating that microorganisms with a similar GC content have similar genome signature patterns [25].

Phylogenetic analysis according to genome signature comparison is not based on multiple alignment data. Thus, bootstrap analysis cannot be performed. In this paper, we estimated the similarity between 2 different tetranucleotide frequencies by using correspondence analysis. The correspondence analysis showed that the genome signature of *D. radiodurans* is most similar to that of *Pseudomonas aeruginosa* (Table 1), whereas the genome signature of *T. thermophilus* is most similar to that of *Th. acidaminovorans* (Table 2). Although the *D. radiodurans* genome signature has similarity to 18 bacterial species within the distance 0.5, the *T. thermophilus* genome signature has similarity only to *Th. acidaminovorans* within the same distance (Table 2). These results indicate that *T. thermophilus* has a different genome signature from those of bacteria included in the high-GC-content cluster (Figure 1).

**Figure 2:**
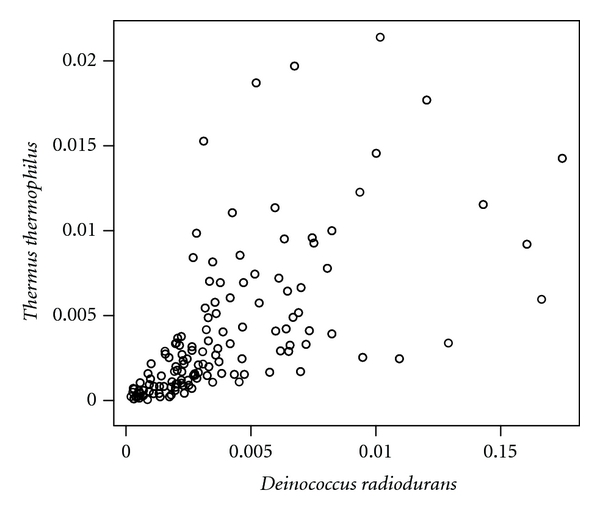
Scatter plot between the tetranucleotide frequencies of the genomes of *Deinococcus radiodurans* and *Thermus thermophilus*.

**Figure 3:**
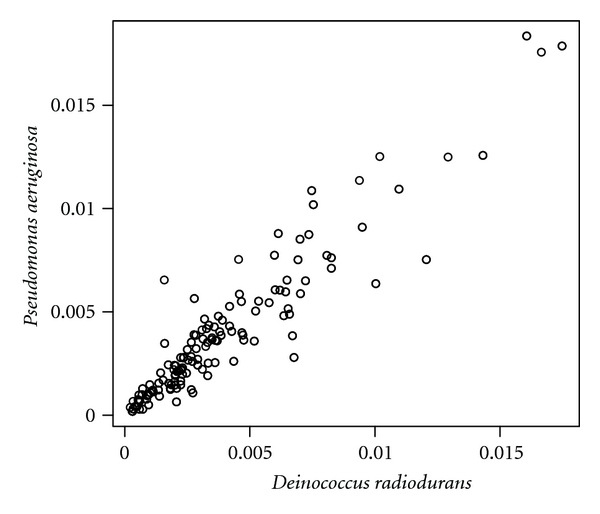
Scatter plot between the tetranucleotide frequencies of the genomes of *Deinococcus radiodurans* and *Pseudomonas aeruginosa*.

**Figure 4:**
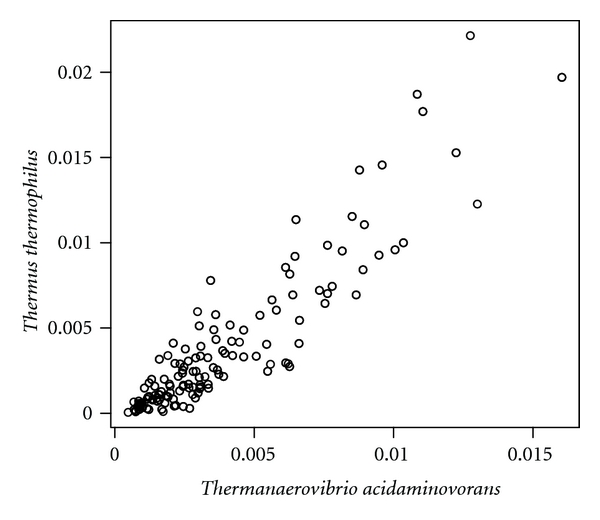
Scatter plot between the tetranucleotide frequencies of the genomes of *Thermanaerovibrio acidaminovorans* and *Thermus thermophilus*.

 Although Pearson's correlation coefficient between the tetranucleotide frequencies of genomes of *D. radiodurans* and *T. thermophilus* is 0.630 (Figure 2), that between the tetranucleotide frequencies of genomes of *D. radiodurans* and *Pseudomonas aeruginosa* is 0.935 (Figure 3) and that between the tetranucleotide frequencies of genomes of *Th. acidaminovorans* and *T. thermophilus* is 0.914 (Figure 4). These results support the results of the neighbor-joining and correspondence analyses.

The frequency of horizontal gene transfer between different bacteria may be associated with genome signature similarity. However, the tree topology based on genome signature (Figure 1) is different from that based on gene content [8]. This is caused by, among others, an amelioration of the horizontally transferred genes [26]. Our findings strongly support the previous report that *Deinococcus* has acquired genes from various other bacteria to survive different kinds of environmental stresses, whereas *Thermus* has acquired genes from thermophilic bacteria to adapt to high-temperature environments [3].

## Figures and Tables

**Table 1:** Distance between *Deinococcus radiodurans* and each bacterium using correspondence analysis.

Bacterial species	Distance
*Pseudomonas aeruginosa *PO1	0.297932379
*Myxococcus xanthus*	0.305390764
*Azorhizobium caulinodans*	0.308895493
*Ralstonia solanacearum*	0.309212661
*Gloeobacter violaceus*	0.317496648
*Symbiobacterium thermophilum*	0.324422553
*Thermomonospora curvata*	0.347077134
*Opitutus terrae*	0.376683191
*Acidobacterium capsulatum*	0.378916616
*Gemmatimonas aurantiaca*	0.383939504
*Rhodobacter sphaeroides *2.4.1	0.386383492
*Rhodospirillum rubrum*	0.392789705
*Streptomyces griseus*	0.415746597
*Geobacter sulfurreducens*	0.425877427
*Agrobacterium tumefaciens*	0.457788385
*Thermomicrobium roseum*	0.460897799
*Syntrophobacter fumaroxidans*	0.470005872
*Sphingomonas wittichii*	0.478630032
*Desulfohalobium retbaense*	0.50752939
*Heliobacterium modesticaldum*	0.512911658
*Chloroflexus aurantiacus*	0.53688488
*Pirellula staleyi*	0.540489386
*Desulfatibacillum alkenivorans*	0.618176651
*Pelotomaculum thermopropionicum*	0.636637282
*Moorella thermoacetica*	0.637983756
*Xylella fastidiosa *9a5c	0.655118109
*Escherichia coli *K-12 MG1655	0.671407958
*Neisseria meningitidis *MC58	0.679417806
*Thermanaerovibrio acidaminovorans*	0.707497366
*Nitrosomonas europaea *ATCC 19718	0.718956013
*Fibrobacter succinogenes*	0.773393097
*Dehalococcoides ethenogenes*	0.793600646
*Vibrio cholerae *N16961	0.794460696
*Desulfitobacterium hafniense *DCB-2	0.823845007
*Thermus thermophilus*	0.831109438
*Shewanella oneidensis*	0.84848937
*Alteromonas macleodii*	0.881823764
*Aminobacterium colombiense*	0.889238858
*Thermobaculum terrenum*	0.899243716
*Syntrophomonas wolfei*	0.90576094
*Bacillus subtilis*	0.913613719
*Coprothermobacter proteolyticus*	0.923779953
*Chlorobium chlorochromatii*	0.926043337
*Coxiella burnetii *RSA 493	0.929681834
*Thermotoga maritima*	0.952651677
*Bacteroides thetaiotaomicron*	0.958944885
*Denitrovibrio acetiphilus*	0.966489936
*Kosmotoga olearia*	0.998958025
*Carboxydothermus hydrogenoformans*	1.012583789
*Nostoc *sp. PCC 7120	1.014447775
*Aquifex aeolicus*	1.03027576
*Chlamydia trachomatis *D/UW-3/CX	1.041383827
*Elusimicrobium minutum*	1.06077929
*Haemophilus influenzae *Rd KW20	1.084974973
*Veillonella parvula*	1.10092918
*Helicobacter pylori *26695	1.124775019
*Cyanothece *sp. ATCC 51142	1.126779861
*Thermoanaerobacter tengcongensis*	1.139238445
*Halothermothrix orenii*	1.149150516
*Eubacterium eligens*	1.164829099
*Natranaerobius thermophilus*	1.167863816
*Prochlorococcus marinus *CCMP1375	1.174664974
*Fervidobacterium nodosum*	1.195233916
*Caldicellulosiruptor saccharolyticus*	1.19880562
*Caldicellulosiruptor bescii*	1.199097055
*Persephonella marina*	1.209862481
*Leptospira interrogans *serovar lai 56601	1.221066506
*Anaerococcus prevotii*	1.224535688
*Petrotoga mobilis*	1.231307366
*Thermodesulfovibrio yellowstonii*	1.242134666
*Trichodesmium erythraeum*	1.246593564
*Sebaldella termitidis*	1.270114395
*Dictyoglomus turgidum*	1.29240584
*Dictyoglomus thermophilum*	1.297069077
*Thermosipho melanesiensis*	1.324630145
*Deferribacter desulfuricans*	1.331638037
*Clostridium acetobutylicum*	1.357082068
*Mycoplasma genitalium*	1.360597739
*Campylobacter jejuni *NCTC 11168	1.374681774
*Leptotrichia buccalis*	1.383345312
*Rickettsia prowazekii*	1.426681449
*Borrelia burgdorferi *B31	1.431569209
*Candidatus *Phytoplasma asteris	1.471567529
*Mesoplasma florum*	1.477622916
*Fusobacterium nucleatum*	1.487576702
*Brachyspira hyodysenteriae*	1.517447262
*Streptobacillus moniliformis*	1.535004291
*Ureaplasma parvum *ATCC 700970	1.559892696

**Table 2:** Distance between *Thermus thermophilus* and each bacterium using correspondence analysis.

Bacterial species	Distance
*Thermanaerovibrio acidaminovorans*	0.468763255
*Symbiobacterium thermophilum*	0.686400076
*Geobacter sulfurreducens*	0.756754453
*Myxococcus xanthus*	0.772836176
*Streptomyces griseus*	0.786527308
*Thermomonospora curvata*	0.791039191
*Moorella thermoacetica*	0.806329416
*Syntrophobacter fumaroxidans*	0.825184063
*Deinococcus radiodurans*	0.831109438
*Desulfohalobium retbaense*	0.835469081
*Rhodospirillum rubrum*	0.836862939
*Azorhizobium caulinodans*	0.837497899
*Gloeobacter violaceus*	0.847382695
*Rhodobacter sphaeroides *2.4.1	0.857474011
*Desulfatibacillum alkenivorans*	0.876877944
*Heliobacterium modesticaldum*	0.886943785
*Pseudomonas aeruginosa *PO1	0.902403886
*Pelotomaculum thermopropionicum*	0.910464775
*Acidobacterium capsulatum*	0.940977424
*Thermomicrobium roseum*	0.958396462
*Agrobacterium tumefaciens*	0.993864461
*Gemmatimonas aurantiaca*	0.993867563
*Ralstonia solanacearum*	0.99540692
*Opitutus terrae*	1.014357577
*Sphingomonas wittichii*	1.018425039
*Chloroflexus aurantiacus*	1.027585883
*Pirellula staleyi*	1.047176443
*Desulfitobacterium hafniense *DCB-2	1.051272244
*Dehalococcoides ethenogenes*	1.071801398
*Xylella fastidiosa *9a5c	1.080146527
*Thermobaculum terrenum*	1.103102039
*Aminobacterium colombiense*	1.103447745
*Syntrophomonas wolfei*	1.119525557
*Nitrosomonas europaea *ATCC 19718	1.125942985
*Escherichia coli *K-12 MG1655	1.136087269
*Neisseria meningitidis *MC58	1.137392967
*Fibrobacter succinogenes*	1.147727362
*Aquifex aeolicus*	1.154770307
*Thermotoga maritima*	1.163190235
*Coprothermobacter proteolyticus*	1.187035315
*Vibrio cholerae *N16961	1.194131544
*Carboxydothermus hydrogenoformans*	1.202997317
*Shewanella oneidensis*	1.207081448
*Bacillus subtilis*	1.236980427
*Coxiella burnetii *RSA 493	1.237627206
*Kosmotoga olearia*	1.240198963
*Alteromonas macleodii*	1.241401986
*Bacteroides thetaiotaomicron*	1.250498401
*Chlamydia trachomatis *D/UW-3/CX	1.259097769
*Chlorobium chlorochromatii*	1.264256111
*Denitrovibrio acetiphilus*	1.264320363
*Nostoc *sp. PCC 7120	1.283892849
*Halothermothrix orenii*	1.307140057
*Thermoanaerobacter tengcongensis*	1.321852789
*Elusimicrobium minutum*	1.327006319
*Cyanothece *sp. ATCC 51142	1.338924672
*Helicobacter pylori *26695	1.353623157
*Veillonella parvula*	1.366604516
*Natranaerobius thermophilus*	1.374016605
*Persephonella marina*	1.384851067
*Prochlorococcus marinus *CCMP1375	1.392425502
*Haemophilus influenzae *Rd KW20	1.392980033
*Anaerococcus prevotii*	1.394012634
*Eubacterium eligens*	1.420199298
*Dictyoglomus turgidum*	1.42068199
*Caldicellulosiruptor saccharolyticus*	1.428805275
*Caldicellulosiruptor bescii*	1.430940559
*Dictyoglomus thermophilum*	1.432160811
*Petrotoga mobilis*	1.43247619
*Fervidobacterium nodosum*	1.436232766
*Leptospira interrogans *serovar lai 56601	1.445508054
*Thermodesulfovibrio yellowstonii*	1.445636432
*Trichodesmium erythraeum*	1.459523665
*Sebaldella termitidis*	1.491593819
*Thermosipho melanesiensis*	1.522817305
*Deferribacter desulfuricans*	1.541728701
*Clostridium acetobutylicum*	1.553667164
*Mycoplasma genitalium*	1.586376378
*Campylobacter jejuni *NCTC 11168	1.590027263
*Leptotrichia buccalis*	1.598390503
*Borrelia burgdorferi *B31	1.626448618
*Rickettsia prowazekii*	1.653875547
*Candidatus *Phytoplasma asteris	1.673704846
*Fusobacterium nucleatum*	1.674099107
*Mesoplasma florum*	1.701326765
*Streptobacillus moniliformis*	1.715886446
*Brachyspira hyodysenteriae*	1.717967185
*Ureaplasma parvum *ATCC 700970	1.784252531
